# The *hector* G-Protein Coupled Receptor Is Required in a Subset of *fruitless* Neurons for Male Courtship Behavior

**DOI:** 10.1371/journal.pone.0028269

**Published:** 2011-11-30

**Authors:** Yuanli Li, Valbona Hoxha, Chamala Lama, Bich Hien Dinh, Christina N. Vo, Brigitte Dauwalder

**Affiliations:** Department of Biology and Biochemistry, University of Houston, Houston, Texas, United States of America; Columbia University, United States of America

## Abstract

Male courtship behavior in *Drosophila melanogaster* is controlled by two main regulators, *fruitless* (*fru*) and *doublesex* (*dsx*). Their sex-specific expression in brain neurons has been characterized in detail, but little is known about the downstream targets of the sex-specific FRU and DSX proteins and how they specify the function of these neurons. While sexual dimorphism in the number and connections of *fru* and *dsx* expressing neurons has been observed, a majority of the neurons that express the two regulators are present in both sexes. This poses the question which molecules define the sex-specific function of these neurons. Signaling molecules are likely to play a significant role. We have identified a predicted G-protein coupled receptor (GPCR), CG4395, that is required for male courtship behavior. The courtship defect in the mutants can be rescued by expression of the wildtype protein in *fru* neurons of adult males. The GPCR is expressed in a subset of *fru*-positive antennal glomeruli that have previously been shown to be essential for male courtship. Expression of *4395-RNAi* in *GH146* projection neurons lowers courtship. This suggests that signaling through the CG4395 GPCR in this subset of *fru* neurons is critical for male courtship behavior.

## Introduction

One critical aspect of understanding how complex behaviors are regulated is to understand the circuits and molecules that are required to generate and display the behavior. Sexual behavior offers an excellent model for the study of complex behaviors because it is both hard-wired and responsive to environmental cues. Courtship behavior in male fruit flies consists of well-defined courtship steps that can easily be studied to examine the effect of mutants.

Sex-specific behaviors in *Drosophila* are under the control of the same master upstream regulators that also regulate somatic sex [1], [2], [3], [4], [5]. They control the sex specific generation of two key male regulators, *fruitless* (fruM) and *doublesex* (dsxM). The male-specific fruM and dsxM proteins are both critically required for normal male courtship behavior [6], [7], [8], [9]. Detailed insight has been gained into the expression pattern of these genes and their regulation by the sex-determination mechanisms, and details are starting to emerge on the sex-specific circuits these neurons may form. While an increasing number of differences are being discovered between males and females in the number of *fru* and *dsx* neurons and the connections they make, it is also evident that many of the neurons that express *fruM* and *dsxM* and form the male circuits are not absent in females [6], [7], [8], [9], [10], [11], [12], [13], [14], [15], [16], [17], [18], [19], [20]. Thus, it is likely that it is the expression of sex specific molecules in these neurons, or the sex-specific nature of their input and/or output that is critical for the observed sex specific behavior. Exploring which genes are expressed in these neurons and how this determines their function and their connections is therefore a critical next step in trying to understand how mating behavior is regulated. Both Fru and Dsx are transcription factors that in microarray studies have been shown to control a large number of genes [21], [22]. However, little is known to date about the significance of these genes, in which cells they might be expressed and what biological role they might play. Given the crucial importance of communication among neurons in circuits, we hypothesized that signaling molecules play an important role in the function of male specific circuits. We describe here the identification of a novel putative G-protein coupled receptor (GPCR), encoded by the gene CG4395, that is required for male courtship. Mutants for the gene, which we named *hector (hec)*, have courtship defects that can be rescued by expression of the wildtype protein in *fru* neurons. Co-localization studies of *fru* and *hec* suggest that the 4395 GPCR is required in a subset of glomeruli in the antennal lobes.

## Results

### CG4395 mutants have reduced male courtship

**Figure 1:**
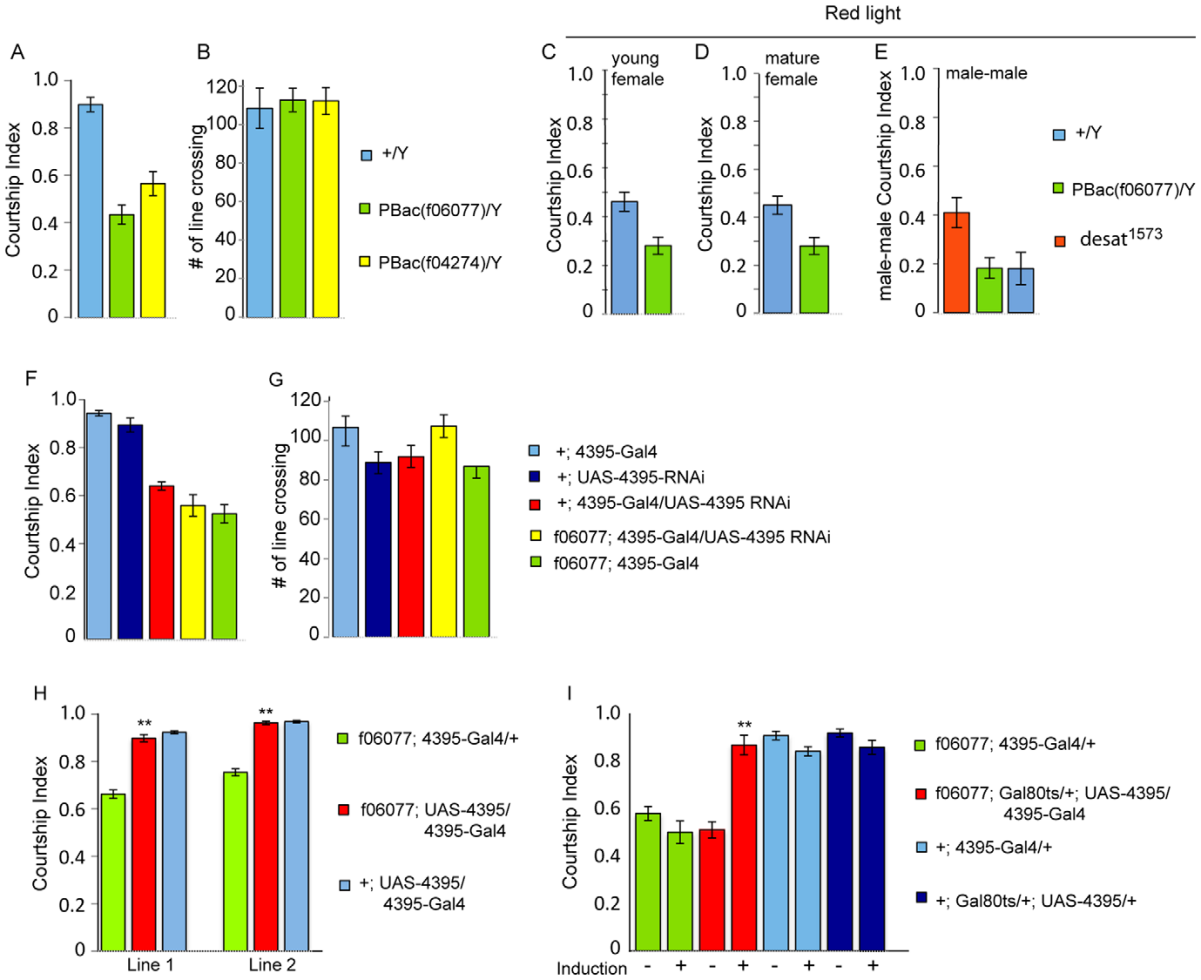
Mutations in the CG4395 GPCR reduce male courtship. Graphs show the courtship index CI (fraction of time males spend courting during the observation period) ± SEM of the indicated genotypes, or the performance of males in a control activity assay (# of line crossings ± SEM). N = 10. Data were analyzed by ANOVA followed by Tukey Kramer multiple comparisons. * p<0.001). CG4395 mutants *PBac(f06077)* and *PBac(f04274*) have a reduced CI in comparison to the control (A), but no defects in activity (B). A similar reduction in courtship is also observed when the assays are performed in red light with either a young or aged virgin female (C, D). Mutant males do not court other males, in contrast to desat1^1537^ males (E). Expression of 4395-RNAi using the promoter construct 4395-Gal4 reduces the CI to the levels observed in the PBac mutants in a wildtype as well as a 4395 mutant background (F); activity in the mutants is not different from the control (G). The *PBac(f06077)* mutant phenotpye can be rescued by expressing wildtype 4395 using the *4395-Gal4* driver and two independent *UAS-4395* responder lines (lines 1 and 2) (H). (I) Conditional rescue in adult males. *UAS-4395* expression is restricted by the presence of *tubP-Gal80^ts^* at 18°C (induction −). Placement of 5 day old males at 32°C for 40 hours (induction +) releases the inhibition and leads to expression of *4395*. Adult induction of *4395* leads to the rescue of the mutant phenotype.

To examine whether CG4395 has a role in male courtship behavior, we tested two different pBac insertions, *PBac(f06077)* and *PBac(f04274)*. CG4395 is located at 11D4. The insertions are located in the last intron of the gene, 4 bp apart, but in different orientations. In order to control for genetic background, we outcrossed the mutants to a cantonized *w^1118^* strain for ten generations. Since the gene is located on the X chromosome, we crossed the mutants to Canton-S wildtype in reciprocal crosses and tested male progeny with or without the mutant chromosome. We observed a significant drop in the courtship index (CI) for both mutants (Figure 1A). The CI expresses the fraction of time a male spends performing any of the steps of the courtship ritual during the observation period. Although courtship of the mutant males is quantitatively reduced, it is not absent. To assess whether particular steps of courtship are affected in the mutants, we quantified latency (time to first orientation towards the female), fraction of wing extension during the overall courtship time, and the number of attempted copulations (Table 1). Mutant males are capable of all steps of courtship but perform them less frequently and seem to lack “motivation” to court. Control short-term activity assays did not show a difference among the genotypes tested (Figure 1B), indicating that the observed courtship phenotype was not caused by general sluggishness or sickness. To further assess the mutant phenotype, we performed additional courtship assays in red light (Figure 1C–E). Under these conditions, pheromonal input plays a major role in mate recognition. Without the visual input, courtship indices are generally lower, also in the control flies. The mutants show a similarly reduced defect in comparison to control males as in white light (Figure 1C). The same is true when they are paired with a matured virgin female (Figure 1D) (young virgin females were used in the other courtship assays). To further examine the effect of the mutation on male courtship behavior, we tested male-male courtship. Mutant males did not exhibit increased male-male courtship when compared to wildtype males and desat^1573^ mutant males whose male-male courtship phenotype has previously been described [23] (Figure 1E). Thus, mutant males appear to be able to distinguish between males and females.

**Table 1:** Analysis of courtship elements in the mutants.

	Latency [s]	Wing Extension (% time of total courtship)	Attempted copulations
*+/Y* (1)	3.8±0.75	0.69±0.06	7.5±2.0
*PBac4395^f04274^*	4.2±0.82	0.36±0.06 *	5.2±1.5
*+/Y* (2)	5.2±0.75	0.65±0.04	5.6±0.9
*PBac4395^f06077^*	5.3±1.3	0.40±0.06 *	3.1±0.9

Individual courtship steps in a standard courtship assay were analyzed for males of the indicated genotypes paired with a wild-type virgin female. Values are mean ± SEM (N = 10). Latency: The time to first orientation toward the female is indicated. For wing extension, the relative time engaged in this behavior relative to the total time spent courting was calculated. For attempted copulation, the total number of events is given. Copulation was not scored since the females were only a few hours old and resisted copulation.

*Values that were significantly different from those of the control flies.

To ascertain that the mutant phenotype we observed is due to the disruption of the 4395 gene, we performed rescue experiments by expressing the wildtype gene in the mutants using the *Gal4/UAS* system. We created *4395-Gal4* transgenic flies by placing 4.7 kb of the 4395 promoter region upstream of Gal4 coding sequences. This fragment contains 1.2 kb upstream of exon 1, the non-coding exon 1, intron 1 and exon 1 right up to the translation start. *UAS-4395* transgenic lines were produced by insertion of the 4395 ORF downstream of UAS. *PBac(f06077)* mutant males carrying 4395-Gal4 and UAS-4395 were tested in courtship. The two independent *UAS-4395* lines tested were capable of completely rescuing the mutant phenotype (p<0.001) (Figure 1H). When *4395-Gal4* was used to express a *UAS-4395-RNAi* transgene we observed a reduction in courtship that was indistinguishable from the *PBac(f06077)* mutants, and which could not be lowered further when *4395-RNAi* was expressed in a *PBac(f06077)* mutant background (p<0.001). Locomotion activity was not affected in these mutants either (Figure 1 F, G). We conclude from these experiments that the *CG4395* GPCR, which we named “*hector* (*hec*)”, is required for male courtship behavior. We next asked whether *hector* is required during development or whether it has a physiological role in adult courting males. We used the *Gal80^ts^* conditional expression system [24] to examine whether expression of *hector* only in adult mature males was capable of rescuing the mutant phenotype. At 18°C, *Gal80^ts^* represses *4395-Gal4*. Thus, *UAS-4395* is not expressed. Upon transfer of the flies to 32°C, *Gal80^ts^* is inactivated, *4395-Gal4* is active and leads to the expression of the *UAS-4395* rescue transgene. Virgin males grown at 18°C were collected and aged individually at 18°C for seven days. They were then placed at 32°C for two days, allowed to adjust to room temperature for one hour and tested for courtship. Control animals were continuously kept at 18°C, allowed to adjust to room temperature for one hour, and tested in parallel to induced flies. We observed complete rescue in flies in which 4395 was induced in adult males (p<0.001), indicating that the gene plays a physiological, rather than a developmental, role in normal courtship (Figure 1 I). This rescue was dose-dependent, as induction for one day only led to partial rescue (data not shown).

### CG4395 is expressed in several brain areas

**Figure 2:**
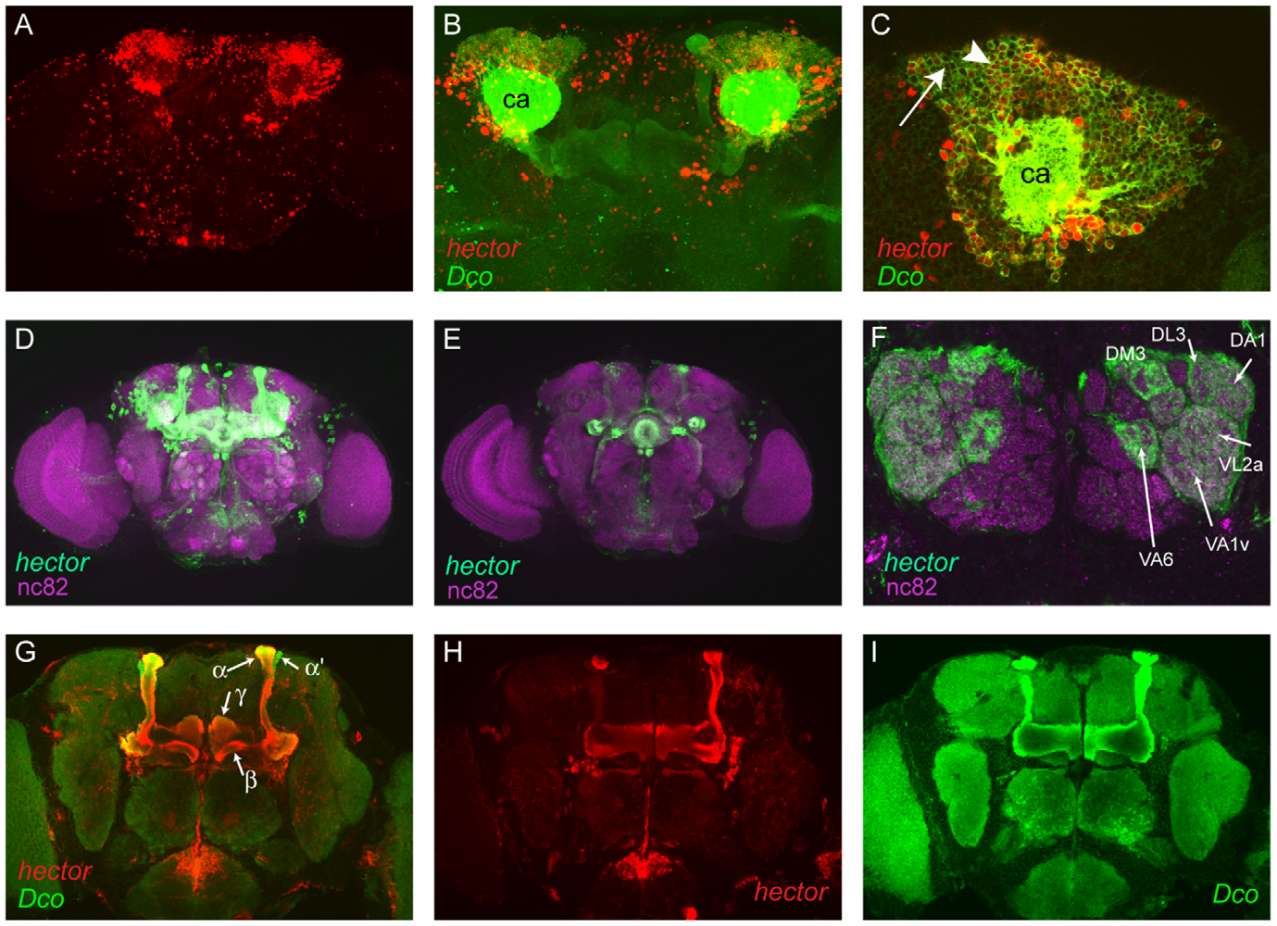
CG4395-Gal4 expression in male brains. (A). Z-stack of anti-β-Gal staining of isolated male brains of *4395-Gal4/UAS-lacZ^nls^*. (B,C). Brains of *4395-Gal4/UAS-lacZ^nls^* males were double-stained with anti-β-Gal (red) and anti-Dco (green). Dco is present in the cytoplasm of the cell bodies of the mushroom bodies and projections of the mushroom bodies [24]. Optical sections are shown. Anti-β-Gal staining is seen in a number of mushroom body cell bodies (arrowhead), but absent from others (arrow). A magnification of (B) is shown in (C). (D–I). Brains of *4395-Gal4/UAS-mcD8::GFP* males were double-stained with anti-GFP (green) and nc82 to visualize neurons (magenta). Projections can be seen in the mushroom bodies (D, Z-stack), the central complex (E, Z-stack) and a subset of glomeruli in the antennal lobes (F, selected optical section). Staining was observed in glomeruli DA1, VA1v, VA1d, VA6, DM3 and DL3 (F). (G–I) To visualize mb projections, double staining was performed with anti-GFP and anti-Dco (G, merged), the single channels are shown in (H) and (I); Z stacks are shown. Abbreviations: (nls) nuclear localization signal; (mcD8::GFP) membrane bound GFP that visualizes neuronal extensions. (mb) mushroom bodies; (ca) calyx of the mushroom bodies; α, β γ: α, β, γ lobes of the mushroom bodies.

To establish where *hector* is expressed, we crossed *4395-Gal4* flies to *UAS-lacZ^nls^* and *UAS-GFP^nls^* reporter lines in which the reporters are located to the nucleus, and examined expression by immunohistochemistry. We observed fairly widespread expression in adult male brains. Prominent expression was observed in what appeared to be mushroom body (mb) cell bodies (Figure 2A). To further characterize this expression, we performed colocalization of 4395-Gal4 driven β-Gal with Dco, the catalytic subunit of PKA which is preferentially expressed in mushroom bodies [25]. As previously reported, Dco is present in mb cell bodies and processes. We observed co-staining in a subset of Kenyon cells. However, *hector* is only expressed in a subset of the *Dco* expressing cells, and not all *4395-Gal4* expressing cells in the dorsal brain area are mushroom body cells (Figure 2B, C).

To further visualize the mushroom body neurons that express *4395-Gal4*, we crossed *4395-Gal4* to *UAS-mcD8::GFP*, thus expressing membrane bound GFP in *hector* neurons. GFP was visualized by anti-GFP antibody staining and co-localization with Dco was examined. We observed expression of *4395-Gal4* in the α, β and γ lobes of the mushroom bodies, but not the α′ and β′ lobes (Figure 2 D, G–I)). Consequently, *4395-Gal4* expressing neurons form a subset of the neurons seen in the peduncle. *4395-Gal4* neurons were further found to project to the wheel of the mushroom bodies and make additional contacts with mushroom body neurons. In addition to expression in the mushroom bodies, prominent labeling of central complex neurons was observed (Figure 2 E). Expression was observed in the ellipsoid body (mainly in the interior layers R1 and R2, but not R4), the fan-shaped body, the noduli and the lateral triangles, the dendrites of ellipsoid body neurons, as well as the protocerebral bridge. Additional expression is visible in the median bundle and a number of isolated larger cells. 4395-Gal4 projections were also observed in a subset of glomeruli in the antennal lobes. Staining was present in the glomeruli DM3, DL3, DA1, VA2a, VA1v and V6 (Figure 2 F). Labeled cell bodies close by suggest that the *4395-Gal4* neurons might be projection neurons.

### *hector* is required in a subset of *fru* cells

**Figure 3:**
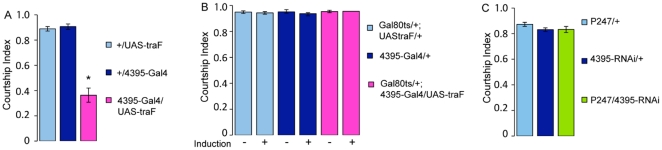
The sexual identity of 4395 Neurons is important for courtship. (A, B) Feminization of *4395-Gal4* expressing cells by the expression of *UAS-traF* reduces male courtship (A), indicating that these cells are sexually dimorphic. In contrast, conditional feminization in adult 5-day old males does not affect male courtship (B). (C). Expression of *4395-RNAi* in the mushroom bodies using the *P247-Gal4* driver does not affect courtship. N = 10.

Which of the cells expressing *4395-Gal4* might be the relevant cells for the role of *hector* in male courtship? While *4395-Gal4* expression in the mushroom bodies is prominent, it has previously been shown that the mb are not required for courtship behavior *per se* [26]. We confirmed this by expressing *4395-RNAi* in the mb using the well-characterized mb-driver P247-Gal4. Like *4395-Gal4*, *P247* is predominantly expressed in the α, β and γ lobes [27]. As expected, these males did not show courtship defects (Figure 3C).

The central complex is implicated in the control of locomotion. Since the *hec ^f06077^* and *hec^f04274^* mutants are normal in an activity assay and can perform all steps of courtship, a reduction of *hector* in the central complex is probably not a major contributor to the courtship defects we observed.

The glomeruli of the antennal lobe in which 4395-Gal4 projections are found include the glomeruli that have been shown to contain *fru* expressing projection neurons (DA1, VA1v, VL2a and V6) and have been implicated in male courtship [28], [29]. They may therefore represent *hector* expressing cells within the antennal lobe that are important for male mating behavior.

Is the sexual identity of *4395-Gal4* expressing cells important for male courtship behavior? We used *4395-Gal4* to express *traF*, the key female regulator protein, to selectively feminize these cells in an otherwise normal male [30]. Males with feminized *4395-Gal4* cells had a significant decrease in their courtship index (Figure 3A). Conditional feminization only in adult flies did not affect male courtship, indicating that the relevant cells are sexually determined prior to adulthood (Figure 3B).

**Figure 4:**
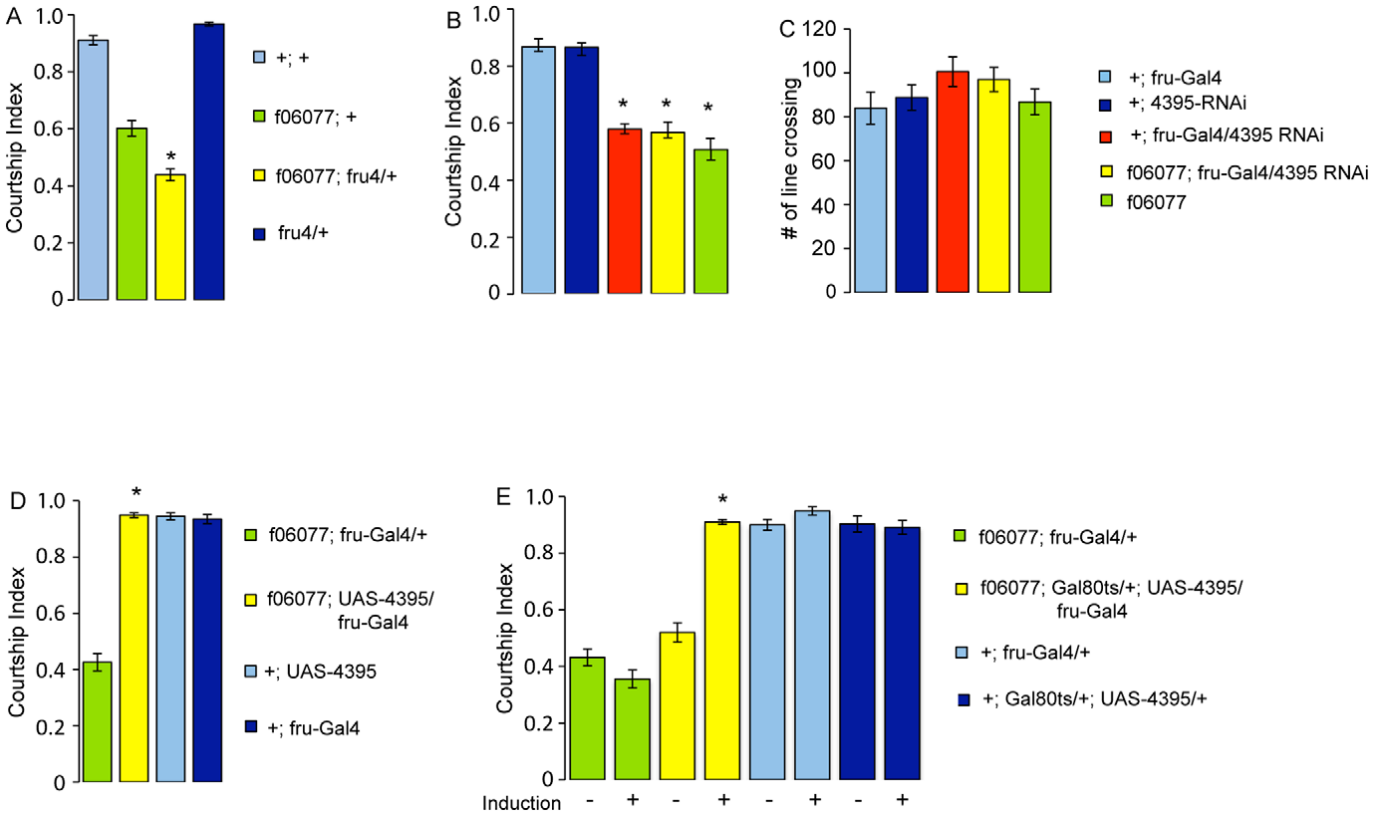
CG4395 is required in *fru* neurons for male courtship. Graphs show the courtship index CI (fraction of time males spend courting during the observation period) ± SEM of the indicated genotypes, or the performance of males in a control activity assay (# of line crossings ± SEM). N = 10. Data were analyzed by ANOVA followed by Tukey Kramer multiple comparisons. * p<0.001). (A) A mutation in *CG4395* and *fru* interact genetically. The CI of *PBac^f06077^; fru^4^/+* males is significantly lower than the CI of *PBac^f06077^* alone. *fru^4^/+* males have a normal CI. (B) Expression of *4395-RNAi* by *fru-Gal4* reduces the CI to the level of the *PBacf^06077^* mutants. The mutants have activity levels that are not different from the controls (C). (D,E) The *PBac^f06077^* mutant phenotype can be rescued by expression of wildtype *UAS-4395* by *fru-Gal4* (D). (E) Conditional adult expression of *UAS-4395* by *fru-Gal4* rescues the mutant phenotype. *UAS-4395* expression is restricted by the presence of *tubP-Gal80^ts^* at 18°C (induction −). Placement of 5 day old males at 32°C for 16 hours (induction +) releases the inhibition and leads to expression of *4395*.

These data demonstrate that the *4395-Gal4* cells that are important for courtship are sexually determined. This finding prompted us to examine the genetic relationship between *fruitless*, a major courtship regulator, and *hector*. When the *hector* mutation was placed in a heterozygous *fru* mutant background, we observed a significantly lower courtship index than in the *hector* mutants alone (p<0.001; Figure 4A). Heterozygous *fru* males do not have courtship defects. This indicates that the two genes are playing a role in the same overall genetic pathway that regulates normal male courtship behavior. This could indicate that *hector* is expressed in *fru* neurons, or/and that the two kinds of neurons interact. To examine the two possibilities, we used *fru-Gal4* to express *4395-RNAi*, thus lowering *hector* in *fru* cells. We found that this led to a reduction of courtship to the levels observed in the *hector* PBac mutants, indicating the importance of signaling through the *hector* GPCR in *fru* neurons (p<0.001), Figure 4B). Again, the additional presence of the PBac^f06077^ *hector* mutation in *fru-Gal4/4395-RNAi* flies did not further lower courtship. The mutants showed normal activity in a short term activity assay (Figure 4C).

To further test the hypothesis that *hector* is a downstream effector in *fru* neurons, we examined whether *hector* mutants could be rescued by the expression of *hec* protein only in *fru* neurons. As shown in Figure 4D, this was indeed the case. Furthermore, like in the case of the rescue with the *4395-Gal4* driver, conditional rescue in adult males using *fru-Gal4* completely rescued the mutant phenotype (Figure 4E). Taken together these findings demonstrate that the *hector* GPCR is required in *fru* neurons of adult males for normal male courtship behavior.

**Figure 5:**
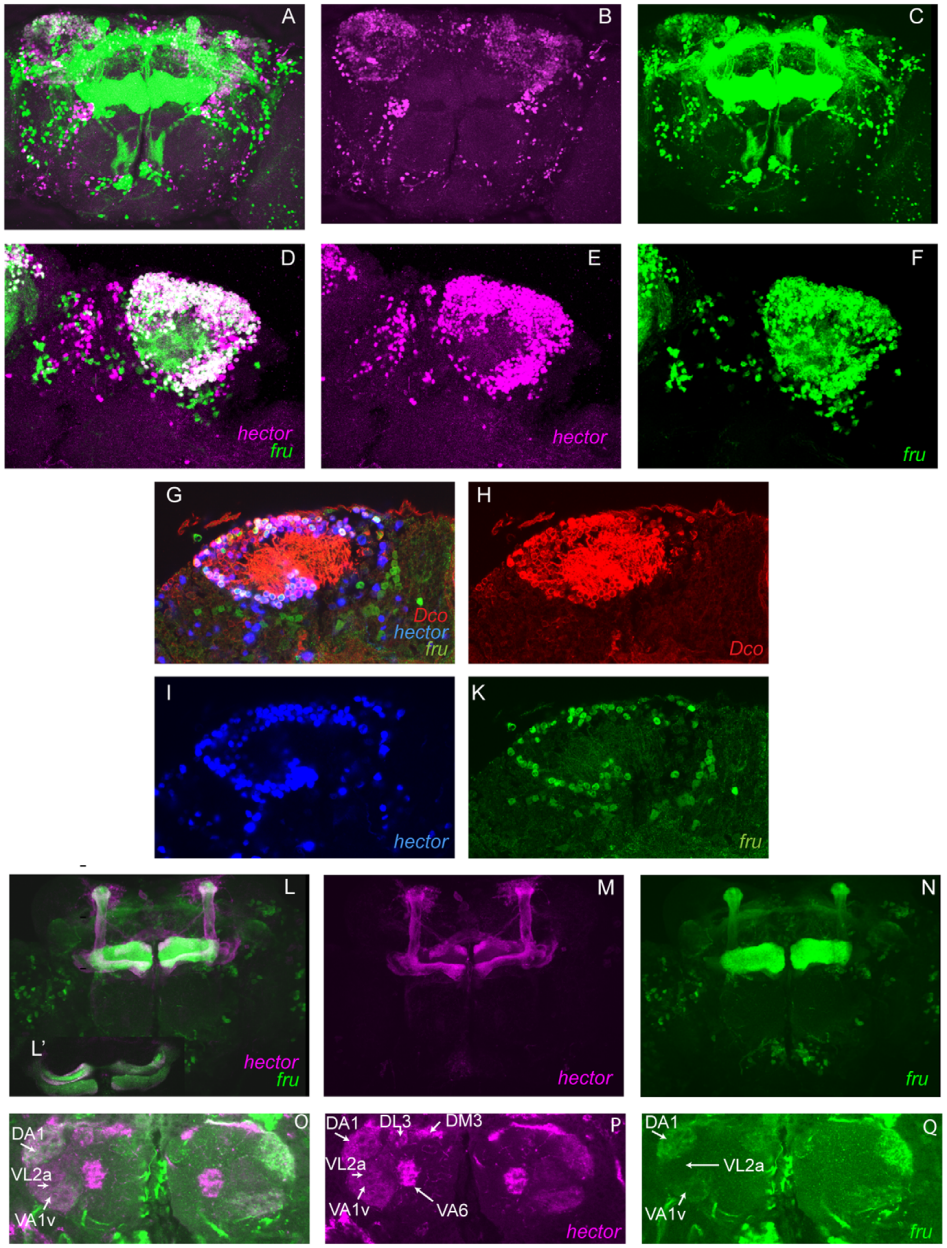
Localization of cells that express both fru and 4395-Gal4. (A–F) Brains of adult *UAS-lacZ^nls^/lexAop::GFP^nls^; 4395-Gal4/fruP1LexA* males were double-stained with anti-β-Gal (magenta) and anti-GFP (green) to identify cells expressing both *fru* and *hector*. Although this GFP carries a nuclear localization signal (nls), diffusion of the protein is also seen into the projections. Co-staining is observed in numerous cell bodies in the mb area. (A, D: merged images, B, C, E, F: single images). A–C show the Z-stack of a whole brain, D–F show a representative magnified optical section in the area of the mb calyx. (G–K) Triple staining of *UAS-lacZ^nls^/lexAop::GFP^nls^; 4395-Gal4/fruP1LexA* males with anti-β-Gal (blue), anti-GFP (green) and anti Dco (red) shows that cells that express both *fru* and *hector* are mushroom body cells. Co-staining is shown in G, single channels are shown in M, N, O. (L–Q) Brains of adult *UAS-mcD8::GFP/4395-Gal4; lexAop-myr-mCherry/fruP1LexA* were co-stained with anti-GFP (magenta) and anti-RFP (green). The projections of the neurons can be seen. In contrast to *4395-Gal4*, *fru*, in addition to expression in the α, β and γ lobes, is also expressed in the α′ and β′ lobes. (L′) shows a magnification at the level of the β and γ lobes that visualizes overlap as well as close proximity of the labeled projections. (O–Q) Colocalization of *fru* and *4395-Gal4* staining in glomeruli of the antennal lobes. 4395-Gal4 projections are visible in all *fru* positive glomeruli.

**Figure 6:**
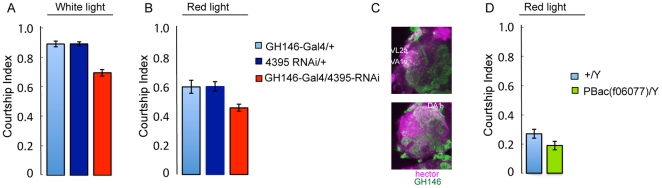
Reduction of *CG4395* RNA in *GH146* neurons reduces courtship. Graphs show the courtship index CI (fraction of time males spend courting during the observation period) ± SEM of the indicated genotypes, N = 10. Expression of *4395-RNAi* in *GH146* projection neurons reduces male courtship in both white and red light (A, B). (C) Optical sections showing co-expression of *UAS-mcD8::GFP/4395-Gal4* (magenta) and *UASQ-tomato::GH146-Q* (green) in antennal glomeruli. (D) *CG4395^f06077^* mutant males missing the third antennal segment court less than control males.

Based on these findings, we suggest that *4395-Gal4* expressing cells that also express *fru* are likely to be the cells that require *hector* for courtship. To identify these cells, we generated flies that express a UAS-reporter under the control of 4395-Gal4 at the same time with a lexAoperator-reporter driven by *fruP1LexA* [*31*]. We chose two kinds of reporters: One set with a nuclear localization signal, and the other expressing membrane-bound reporters in order to visualize neuronal projections. The results are shown in Figure 5. In the mushroom body area, numerous cells express both 4395-Gal4 and fru (Figures 5 A–F). In contrast to 4395-Gal4, *fru* is also expressed in the α′ and β′ lobes (Figures 5 L, L′, M, N). In order to assess the nature of cells that express both *4395-Gal4* and *fru* in the mushroom body area, we performed triple staining with anti-Dco. A representative optical section is shown in Figures 5 G–K. The cells co-expressing *4395-Gal4* and *fru* were found to be mushroom body neurons. We did not observe cells that co-expressed fru and 4395-Gal4 in the central complex. Staining in the antennal lobes was much weaker for both *fru* and *4395*-Gal4 than in the other brain cells. *fru* expression was seen in glomeruli DA1 and VA1v (Figure 5Q), but presumably staining was too weak to see expression in VL2a and VA6, where its expression has also been reported [29]. *hector* expression was observed in the same subset of glomeruli already described in Figure 2 (Figure 5P) . This confirms expression of 4395-Gal4 in *fru+* glomerular neurons of the antenna lobes (figure 5O) and suggests that *hector* may be required for male courtship in these cells. To further examine this possibility, we used *GH146-Gal4* to express *4395-RNAi*. *GH146-Gal4* labels a large subset of projection neurons in the antennal glomeruli. Silencing of *GH146* neurons has been shown to affect courtship and these neurons have been shown to be part of the sexually dimorphic neuronal pathway that responds to cis vaccenyl acetate (cVA) [18], [32], [33]. The *GH146-Gal4/4395-RNAi* males showed significantly reduced courtship in white light as well as red light (Figure 6A, B)). This indicates a role for *CG4395* in a subset of *GH146* projection neurons. However, the effect was not as pronounced as in *fru-Gal4/4395-RNAi* flies, suggesting an additional role for CG4395 outside of GH146 projection neurons, or a different strength of the two drivers. Using a *GH146Q/UASQ-tomato* reporter line, we did find co-staining of *GH146Q* and *CG4395-Gal4* (Figure 6C), as expected from our results. In order to examine whether *CG4395's* main function might be in olfaction, we removed the third antennal segment, which contains the olfactory receptors, in control *CS* flies and in *CG4395* mutant flies and examined their courtship in red light (Figure 6D). Courtship in all flies was reduced, as expected. The CI of the mutant flies was still significantly lower than in wildtype, arguing that CG4395 also has a role outside of olfaction.

## Discussion

Our data demonstrate a role for the CG4395 “*hector*” encoded GPCR in the regulation of *Drosophila* male courtship behavior. While *hector* mutant males are capable of performing all steps of courtship, the probability that they maintain and re-initiate courtship is low, resulting in a significantly reduced courtship index. Our experiments show that the courtship defect can be rescued by conditional expression of the gene in adult males. This demonstrates that the gene is required in adult males for normal courtship, thus indicating a physiological role for the protein in mating behavior. Based on its similarity to GPCRs, the most likely role for *hector* is in signaling processes that mediate the behavior. The *hector* gene codes for an orphan predicted neuropeptide GPCR that is related to the class of secretin-like receptors [34]. These include the mammalian calcitonin gene-related peptide (CGRP) receptors that bind Calcitonin (32aa), Calcitonine-related peptide and Amylin [35]. Among other things, Amyilin has been shown to regulate feeding related changes [36]. Interestingly, *Takeout*, a secreted protein that is required for male courtship has, in addition to its role in courtship, been reported to play a role in feeding behavior in response to starvation [37], [38].

*hector* is expressed in numerous cells in the fly brain. While a number of them remain unidentified, dominant expression is observed in the neurons of three defined brain structures: The mushroom bodies, the central complex and, at lower levels, in a subset of glomeruli in the antennal lobes. A large number of *hector* expressing cells are part of the mushroom body neurons that form the α, β and γ lobes. The mushroom bodies play a central role in learning and memory [39]. However, they have been shown to not be required for basic male courtship behavior [26]. We have confirmed that expression of *hector* in the mushroom bodies is not required for male courtship: Expression of 4395 RNAi by the P247-Gal4, a driver expressed predominantly in the α, β and γ lobes of the mushroom bodies [27], did not affect male courtship, whereas expression of the same *4395-RNAi* by *4395-Gal4* lowered male courtship to the levels observed in the PBac mutants. These data demonstrate that expression of *hector* in the mushroom bodies is not required for male courtship behavior, in agreement with previous findings that these structures are dispensable for basic courtship.

Expression of *hector* is also observed in a number of structures of the central complex. The central complex plays a crucial role in the control of locomotion [40]. However, our short term activity assays which reflect motor behavior did not indicate locomotion defects in the *hector* mutants we examined. Furthermore, we did not observe co-expression of *hector* and *fru* in central complex neurons (see below).

Our initial genetic interaction studies indicated that *hector* and *fru* act in the same overall pathways that regulate male courtship behavior. Remarkably, when we expressed *hector* in *fru* neurons by using the *fru-Gal4* driver, this expression was sufficient to completely rescue the *hector* mutant phenotype, indicating that *hector* is required in *fru* neurons. Furthermore, expression of *4395-RNAi* in *fru* neurons using *fru-Gal4* leads to a mutant phenotype that is indistinguishable from the ones observed in the pBac mutants. This implies a crucial role for *hector* in the control of male courtship behavior in a subset of *fru* neurons that co-express *fru-Gal4* and *4395-Gal4*. When we examined the co-expression of *fru* and *hector* we observed extensive co-labeling in the lateral protocerebrum. Not all *hector* expressing cells also express *fru*, and only a subset of *fru* cells expresses *hector*. Triple labeling experiments with anti-*Dco*, a mushroom body marker [25], demonstrated that the cells in the lateral protocerebrum that express both *fru* and *hector* are mushroom body neurons. As described above, mushroom bodies are not required for male courtship. This implies that cells outside the mushroom bodies, where *hector* and *fru* are co-expressed, are the cells that are required for male courtship.

While we observed a few additional isolated cells that express both, the only other neurons where we have observed *hector* expression where *fru* is also expressed are neurons in a subset of glomeruli in the antennal lobes. Although expression of our reporters in the antennal lobes was much weaker than in other parts of the brain, we have observed *hector* projections in glomeruli DM3, DL3, DA1, VA2a, VA1v and V6. This includes all of the glomeruli that have been shown to be innervated by both *fru* ORN and *fru* projection neurons (DA1, VA1v, VL2a and V6, [28], [29]. The best characterized among them is the DA1 glomerulus. It is innervated by the olfactory receptor neuron Or67d and has been shown to be involved in the perception of 11-cis-vaccenyl acetate (cVA) in both males and females [41], [42], [43]. cVA acts as a repellant in males, and an attractant in females. Or67d mutant males show increased courtship towards other males, but maintain courtship towards females. In contrast to the findings with Or67d mutants, we have not observed male-male courtship in *hector* mutants. We have also not observed an increase in latency, i.e. the time to first orientation towards the female, which is thought to be an indicator of impairment in perceiving pheromonal cues. Significantly, Stockinger et al. (2005) have shown that silencing of all of the *fru* projection neurons in the antennal glomeruli resulted in male-female courtship defects that were as strong as when they silenced all *fru*-expressing neurons [29]. This result implies an important role for these glomeruli in male-female courtship. Given the presence of *hector* projections in these glomeruli, and the results we obtained using *fru-Gal4* driven rescue as well as *hector* knockdown by RNAi, we propose that the phenotypes we observe are in large parts caused by the absence of *hector* signaling in these glomerular *fru* neurons. Hence, signaling through the hector GPCR is likely a crucial component of male courtship in this subset of *fru* neurons. The observed courtship reduction in GH146/4395-RNAi males suggests that some of these neurons are projection neurons.

The *hector* protein is one of the few molecules identified that are specifically and functionally required in *fru* neurons. In support of this, we also find that the feminization of *hector* expressing cells leads to courtship defects, demonstrating the sexually dimorphic nature of these neurons. However, feminization of these neurons in adult males does not affect courtship. This is in agreement with earlier genetic studies that suggested that the basic competence for male courtship is established in late pupal stages [44], [45]. *hector* does not appear to be required to establish the male characteristics of *fru* neurons during development, since conditional expression of hector in adult males using the *4395-Gal4* as well as the *fru-Gal4* driver was capable of completely rescuing the mutant phenotype. These results indicate that *hector* signaling is required to mediate adult male-specific signaling that is required for normal courtship. These signals may come from the *fru* ORN that project to these glomeruli, or from interneurons or other projection neurons. A fairly large number of neurotransmitters and neuropeptides has been found in the antennal lobes [46], and it remains to be seen what the ligand is for *hector*, and what signaling pathways lie downstream.

## Materials and Methods

### Fly strains

All flies strains were reared on standard corn meal/sugar-based medium at room temperature, except for Gal80^ts^ flies that were grown at 18°C and induced as adults at 32°C as indicated. The *CG4395* mutants *PBacCG4395[f04274]* and *PBacCG4395[f06077]* were obtained from the Exelixis *Drosophila* Stock Collection at the Harvard Medical School. *traF* is *w^1118^; P{UAS-tra.F}20J7* [*30*]. *w^1118^(CS)* and w; *GH146-Gal4* were a gift from Gregg Roman, University of Houston. *fru-Gal4* [12] was a gift from Barry Dickson, IMP, Austria. *fruP1.lexA* [31] was a gift from David Mellert and Bruce Baker, Janelia farms. *LexAop-myr Cherry* [47] was a gift from Soeren Diegelmann and Matthias Landgraf, Cambridge and Erin Savner, Columbia University. *UAS-4395 RNAi* is VDRC stock no. 7223. *desat1^1573^* [23] was a gift from Jean-François Ferveur, Université de Bourgogne, Dijon, France.

w^*^; P{tubP-GAL80^ts^}20; TM2/TM6B, Tb^1^(stock no. 7019), *w^1118^; P{UAS-lacZ.NZ}J312* (stock no. 3956), y^1^ w*; P{UAS-mCD8::GFP.L}LL5 (stock no. 5137), *w^1118^; P{UAS-GFP.nls}14*, *w^1118^; lexA-2×hrGFP.nls* (chrs 2, stock no. 29954), *w^1118^; UAS-lacZ,NZ 20b* (chrs 2, stock no. 3955), and *w^1118^; {GH146-QF.P}53 P{QUAS-mtdTomato-3×HA}24A* (stock no. 30037) [48] were obtained from the Bloomington stock center.

### Courtship assay and Short term activity assay

Assays were performed as described [37]. Tested males were 4–6 days old. For experiments with mature virgin females, females were collected 2–3 hours after eclosion and aged in groups for 4 days before the courtship assay. Male–male courtship was performed as described in [23]. For experiments with removed antennae, antennae of newly eclosed virgin males were removed with a razor blade. The flies were then put in individual vials and tested for courtship 4–5 days later.

### Immunohistochemistry

The brains of 4–5 day-old male flies were dissected in 1× PBS and fixed in freshly prepared 4% paraformaldehyde in PBSH (PBS containing 1 M NaCl) for 20 min at room temperature. All subsequent procedures were performed at room temperature, except for antibody incubations. Brains were washed three times in 1× PBHS/0.5% Triton for 15 min each and then washed three times in 0.1 M Tris-HCl/0.3 M NaCl (pH 7.4), containing 0.5% Triton X-100 (TNT) for 15 min each. Blocking was performed in TNT solution containing 4% normal goat serum (blocking buffer) for 1.5 h. The primary antibody was applied in blocking solution and incubated overnight at 10°C. The brains were rinsed six times for 15 min each and five times for 30 min each in TNT. The secondary antibody was diluted in blocking solution and incubated overnight at 10°C. Brains were washed six times for 15 min each. Fluorescently labeled brains were mounted with Vectashield mounting medium (Vector Laboratories, CA). For double-staining experiments, antibody stainings were performed sequentially using the procedure described above.

### Antibodies used

Chicken anti-β-galactosidase (abcam ab9361) 1∶500; Mouse anti-β-galactosidase (Sigma G8021) 1∶100; Rabbit anti-β-Galactosidase; Rabbit anti-RFP (abcam ab62341) 1∶100, Rabbit anti-GFP (Invitrogen 673782) 1∶100; Mouse anti-GFP (Roche 11814460001) 1∶50, Mouse nc82 (Developmental Studies Hybridoma Bank, Univ. of Iowa) 1∶20, Rabbit anti-Dco (Skoulakis et al., 1993) 1∶100, Alexa Fluor 635 goat anti-mouse (Invitrogen A31575) 1∶200, DyLight 405 goat anti-chicken (Jackson Immuno Research 103-475-155) 1∶200, Alexa Fluor 555 goat anti-rabbit (Invitrogen A21429) 1∶200.

### Confocal microscopy

Fluorescent preparations were viewed using an Olympus FV1000 confocal microscope, Images were processed using Adobe Photoshop.

### Generation of the 4395-Gal4 and UAS-4395 transgenes

4395-Gal4: 4.7 kb immediately upstream of the CG4395 translation initiation site, including 1.2 kb of upstream region, plus the non-coding exon 1 and intron 1 were amplified from CS genomic DNA and inserted into the pPTGAL transformation vector [49] as a BamH1/XbaI fragment. The primers used for PCR amplification were 5′-CGGGATCCGTGACTAACTCTTGTCAACGGCTAATGC-3′ and 5′-GCTCTAGA GTTGCCGATGGAACTGATGTCGC-3′, with unique 5′ restriction sites (underlined).

UAS4395: The open reading frame of 4395 was amplified by RTPCR from Canton-S head RNA isolated with RNAzol (Invitrogen). cDNA was prepared using the Superscript II kit (Invitrogen).The fragment was cloned into the pPUAST transformation vector. All constructs were verified by sequencing. Transgenic lines were established by Rainbow Transgenic Flies, Inc., CA, in a *w* ^1118^ background, by P-element mediated transformation.

### Statistical analysis

Analysis of variance (ANOVA) was performed, followed by Tukey Kramer pairwise comparisons using JMP (version 8.0.2 for Windows, SAS Institute, Inc.,). or Instat (version 3 for Macintosh, GraphPad Software , Inc.) software.
